# Predicting the treatment response of certolizumab for individual adult patients with rheumatoid arthritis: protocol for an individual participant data meta-analysis

**DOI:** 10.1186/s13643-020-01401-x

**Published:** 2020-06-12

**Authors:** Yan Luo, Konstantina Chalkou, Ryo Yamada, Satoshi Funada, Georgia Salanti, Toshi A. Furukawa

**Affiliations:** 1 Department of Health Promotion and Human Behavior, School of Public Health in the Graduate School of Medicine, Kyoto University, Yoshida Konoe-cho, Sakyo-ku, Kyoto, 606-8501 Japan; 2 Institute of Social and Preventive Medicine, University of Bern, Bern, Switzerland; 3 Unit of Statistical Genetics, Center for Genomic Medicine, Graduate School of Medicine, Kyoto University, Kyoto, Japan; 4 Department of Urology, Graduate School of Medicine, Kyoto University, Kyoto, Japan

**Keywords:** Rheumatoid arthritis, Certolizumab, Individual participant data meta-analysis, Prediction model, Treatment response

## Abstract

**Background:**

A model that can predict treatment response for a patient with specific baseline characteristics would help decision-making in personalized medicine. The aim of the study is to develop such a model in the treatment of rheumatoid arthritis (RA) patients who receive certolizumab (CTZ) plus methotrexate (MTX) therapy, using individual participant data meta-analysis (IPD-MA).

**Methods:**

We will search Cochrane CENTRAL, PubMed, and Scopus as well as clinical trial registries, drug regulatory agency reports, and the pharmaceutical company websites from their inception onwards to obtain randomized controlled trials (RCTs) investigating CTZ plus MTX compared with MTX alone in treating RA. We will request the individual-level data of these trials from an independent platform (http://vivli.org). The primary outcome is efficacy defined as achieving either *remission* (based on ACR-EULAR Boolean or index-based remission definition) or *low disease activity* (based on either of the validated composite disease activity measures). The secondary outcomes include ACR50 (50% improvement based on ACR core set variables) and adverse events. We will use a two-stage approach to develop the prediction model. First, we will construct a risk model for the outcomes via logistic regression to estimate the baseline risk scores. We will include baseline demographic, clinical, and biochemical features as covariates for this model. Next, we will develop a meta-regression model for treatment effects, in which the stage 1 risk score will be used both as a prognostic factor and as an effect modifier. We will calculate the probability of having the outcome for a new patient based on the model, which will allow estimation of the absolute and relative treatment effect. We will use R for our analyses, except for the second stage which will be performed in a Bayesian setting using R2Jags.

**Discussion:**

This is a study protocol for developing a model to predict treatment response for RA patients receiving CTZ plus MTX in comparison with MTX alone, using a two-stage approach based on IPD-MA. The study will use a new modeling approach, which aims at retaining the statistical power. The model may help clinicians individualize treatment for particular patients.

**Systematic review registration:**

PROSPERO registration number pending (ID#157595).

## Background

Rheumatoid arthritis (RA) is a chronic inflammatory disease, for which we cannot currently expect complete cure. The drugs that can delay disease progression are known as disease-modifying anti-rheumatic drugs (DMARDs). There are 3 categories: conventional synthetic DMARDs (csDMARDs), biologic DMARDs (bDMARDs), and targeted synthetic DMARDs (tsDMARDs). bDMARDs can be further divided into several subtypes according to the target, among which the tumor necrosis factor (TNF) α inhibitors are the most classic and widely used.

Most RA patients undergo long-term treatment. According to the treat-to-target strategy proposed by the EULAR (European League Against Rheumatism) practice guideline [1], repeated assessment of disease activity should be performed every 3 to 6 months after a treatment is given, to evaluate the response and decide the next-step strategy: switching drugs, maintenance, tapering, or discontinuation. Hence, the disease course of RA is composed of many *short-term* (3 to 6 months) intervention-response loops. For the purpose of improving *long-term* prognosis, such as delaying the progression of bone fusion or functional deficiency, *short-term* intervention-response loops need to have beneficial outcomes [2].

To find the *optimal* treatment for a *particular* patient, it is necessary to personalize the treatment. It would be helpful if we could predict the probability of treatment response based on the patient’s genetic, biologic, and clinical features. However, common evidence in the form of randomized controlled trials (RCTs) or their meta-analyses (MAs) at the aggregate level only reports average results. The drug that works for the average patients might not work or even be harmful for a particular patient. Consequently, it is desirable to identify subgroups of patients associated with different treatment effects.

Individual participant data meta-analysis (IPD-MA) has been previously employed to develop prediction models for treatment effects [3–6]. Previous treatment response prediction models for RA were mainly based on *observational studies* [7–11]. Observational studies seem suited for predicting the *absolute risk* of an outcome, but it may be less satisfactory in estimating the *relative risk* between different drugs, because unknown confounders may persist even when we try to adjust for known confounders. On the other hand, though the population in RCTs is highly restricted hence may be less representative, data from RCTs are more rigorously collected and more likely to provide an unbiased estimate of the relative treatment effects [12]. The synthesis of RCT data via IPD-MA can increase the statistical power [13] and have been used to predict treatment response [6, 14–17]. To the best of the authors’ knowledge, such an approach has not been taken to predict treatment response in RA to date.

Our aim is to develop a prediction model of treatment effects based on individual characteristics of RA patients through IPD-MA. Since TNFα inhibitors are the most classic and widely used bDMARDs for RA, we will build a model for certolizumab (CTZ), a TNFα inhibitor with sufficient IPD data, in this study. We will first estimate the pooled average effect sizes for the primary and secondary outcomes using one-stage Bayesian hierarchical IPD-MA. The main objective of the study is to use a two-stage risk modeling approach to predict the individualized treatment effects interest [12]. The first stage is to build a multivariable model aiming to predict the baseline risk for a particular patient blinded to treatment. In the second stage, this baseline risk score will be used as a prognostic factor and an effect modifier in an IPD meta-regression model to estimate the individualized treatment effects of CTZ. We consider to validate and optimize the modeling approach in the present study, and plan eventually to expand it to an IPD network meta-analysis to compare several drug types (e.g., interleukin-6 inhibitors, anti-CD20 antibodies) as our future research perspective.

## Methods

The present protocol has been registered within the PROSPERO database (provisional registration number ID#157595) and is being reported in accordance with the reporting guidance provided in the Preferred Reporting Items for Systematic Reviews and Meta-Analyses Protocols (PRISMA-P) statement [18] (see the checklist in Additional file 1). The proposed IPD-MA will be reported in accordance with the reporting guidance provided in the Preferred Reporting Items for Systematic Reviews and Meta-analyses of Individual Participant Data (PRISMA-IPD) statement [19]. Any amendments made to this protocol when conducting the study will be outlined and reported in the final manuscript.

### Eligibility criteria

Studies will be selected according to the following criteria: patients, interventions, outcomes, and study design.

#### Patients

We will include adults (18 years or older) who are diagnosed with either early RA (2010 American College of Rheumatology (ACR)/European League Against Rheumatism (EULAR) classification criteria) [20, 21] or established RA (1987 classification criteria) [22]. Patients with inner organ involvement (such as interstitial lung diseases), vasculitis, or concomitant other systemic autoimmune diseases will be excluded. We will include both treatment-naïve patients and patients who have insufficient response to previous treatments. We will include patients with moderate to severe disease activity based on any validated *composite* disease activity measures. Patients who have already achieved remission or at low disease activity at baseline will be excluded. Patients who have used certolizumab (CTZ) within 6 months before randomization will be excluded.

#### Interventions

We will include RCTs which compare *certolizumab (CTZ) plus methotrexate (MTX)* with *MTX monotherapy*, regardless of doses. If a study compares *CTZ + any csDMARDs* with *any csDMARDs*, we will only include patients on *CTZ + MTX* or *MTX* from that study. Trials investigating the tapering or discontinuation strategy of CTZ will be excluded.

#### Outcomes

Our primary outcome is efficacy defined by *disease states*, which is achieving either *remission* (based on ACR-EULAR Boolean or index-based remission definition [23]) or *low disease activity* (based on either of the validated composite disease activity measures [24]: DAS28 (Disease Activity Score based on the evaluation of 28 joints) ≤3.2 [25], CDAI (Clinical Disease Activity Index) ≤ 10 [26], SDAI (Simplified Disease Activity Index) ≤ 11 [27]) at 3 months (allowance 2–4 months) after treatment, as a binary outcome. We choose it as our primary outcome because it is suggested as the indicator of the treatment target in both the practice guideline [1] and the guideline for conducting clinical trials in RA [2], and it has been shown to provide more information for future joint damage and functional outcomes compared to relative response (change from baseline) [28].

We have two secondary outcomes. One is efficacy defined by *response* (improvement from baseline), for which we will use the ACR response criteria ACR50 (50% improvement based on ACR core set variables) [29]. Another is adverse events (AEs). We will perform an IPD-MA separately for patients with all kinds of infectious AEs within 3 months since it is one of the most important AEs for biologic agents. We will also describe other noticeable AEs within 3 months reported in the trials. We will not make predictions models for the secondary outcomes.

#### Study design

We will include double-blind RCTs only. If there are crossover RCTs, only the data of the first phase will be used for analysis. Cluster RCTs, quasi-randomized trials, and observational studies will be excluded.

### Information source and literature search

We will conduct an electronic search of Cochrane CENTRAL, PubMed, and Scopus from inception onwards, with the keywords: “rheumatoid arthritis,” “certolizumab” or “CDP870” “Cimzia”, “methotrexate” or “MTX,” without language restrictions. A draft search strategy is provided in Additional file 2. We will search WHO International Clinical Trials Registry Platform to find the registered studies. We will search the US Food and Drug Administration (FDA) reports to see if there are any unpublished reports from the pharmaceutical companies. For IPD, we will contact the company which markets certolizumab and request IPD through http://vivli.org. We will assess the representativeness of the IPD among all the eligible studies by investigating the potential differences between trials with IPD and those without IPD.

### Screening and selection procedure

Two independent reviewers will screen the titles and abstracts retrieved from the electronic searches to assess for inclusion. If both reviewers agree that a trial does not meet eligibility criteria, it will be excluded. The full text of all the remaining articles will be obtained for further reading, and the same eligibility criteria will be applied to determine which to exclude. Any disagreements will be resolved through discussion with a third member of the review team.

### Data collection

#### At aggregate level

Two reviewers will independently extract the information for all the included studies at aggregate level. A detailed data extraction template will be developed and piloted on 3 articles; after finalizing the items on the data extraction form, the 3 articles will be re-extracted. The main information includes intervention/control details, trial implementation features (e.g., completion year, randomized numbers, dropouts, follow-up length), baseline demographic and disease-specific characteristics, and outcomes of interested. The above information will be used for: (1) exploring the representativeness of the trials with IPD among all the eligible trials and (2) confirming if the IPD is consistent with the reported results.

#### At IPD level: for studies with IPD available

When the IPD is ready to be used, we will identify the variables of interest before the analysis. The variables regarding intervention, control, and outcomes are defined as the above in the “Eligibility criteria” section. With regard to *patient or trial characteristics to be used as potential covariates in the prognostic model*, based on the literature [30–32] and our clinical practice, we propose the following factors as candidates of potential *prognostic factors* (PFs, baseline factors that may affect the response regardless of the treatment) (Table 1), which will be used for baseline risk model development (see the “Predicting treatment effect for patients with particular characteristics: a two-stage model” section below). We will try to collect all the information listed in Table 1 from the data, but only available factors that have been recorded in the trials will be added into the model. We will decide in which type (e.g., continuous, categorical, binary, etc.) a covariate will be put into the model according to the distribution of that covariate after we obtain the data.

**Table 1:** Potential candidates to be involved as prognostic factors in the prognostic model

***Demographics***	
Age*, sex*, ethnicities	
BMI*, smoking history*	
***Clinical features***	
Family history of first-degree relatives	
Length of time since first onset until the trial commencement, length of time since first onset until first treatment*	
Disease activity: • Number of tender joints*, number of swelling joints*, self-report level of pain based on visual analogue scale (VAS)* • Disease Activity Score (continuous)*, disease activity level (categorical)*	
Joint involvement: • Large joint involvement: knee, hip joints, etc. • Uncommon joint involvement	
Nonspecific systemic symptoms: fever, fatigue, etc.	
Comorbidities*: osteoporosis, osteoarthritis, etc.	
Functional/global quality of life (QoL) conditions at baseline*	
Prior treatment history: failure times, failed drug types, etc.	
Cointerventions decided before randomization: • Steroids, nonsteroidal anti-inflammatory drug (NSAIDs)	
***Biochemical features***	
Serum inflammatory factors*: • Erythrocyte sedimentation rate (ESR), C-reactive protein (CRP); others such as TNF, IL-6, etc.	
Serum antibodies*: • Rheumatoid factor (RF), anti-cyclic citrullinated peptide (anti-CCP), antinuclear antibody (ANA) spectrum	
***Radiographic features***	
Joint fusion (already deformed), bone erosion*, synovitis*, early bone inflammation	
Radiographic scores*	
***Genetics***^#^	
HLA (human leukocyte antigen) types and SNPs (single nucleotide polymorphisms) if they are tested*.*	

*Factors that have been proved to be a prognostic factor for any treatments in previous studies

^#^Since genetic tests for RA are not routinely implemented in clinical practice, we anticipate that most studies will not report them. Although genetics are often considered critical in precision medicine, we will consider it justifiable if no genetic information is included in our model, because there is no single one that has been proven to be strongly associated with the prognosis or treatment responses, and two studies have indicated that genetic information barely contribute in predicting treatment effects [33]

### Risk of bias assessment

Two independent reviewers will assess the risk of bias (RoB) for each included RCT according to “RoB 2 tool” proposed by the Cochrane group [34]. For the efficacy primary outcome, RCTs will be graded as “low risk of bias,” “high risk of bias,” or “some concerns” in the following five domains: risk of bias arising from the randomization process, risk of bias due to deviations from the intended interventions, missing outcome data, risk of bias in measurement of the outcome, and risk of bias in selection of the reported result. The assessment will be adapted for IPD-MA, i.e., as per the obtained data and not the conducted and reported analyses in the original publications. Finally, they will be summarized as an overall risk of bias according to the RoB 2 algorithm.

### Data analysis

Since our primary aim is to develop a prediction model and not to get a precise estimation of the treatment effects, all the analyses will be based on IPD only. Therefore, we will neither analyze aggregate data together nor investigate the robustness of the IPD-MA including aggregate data, for they are beyond the perspectives of the present study.

#### Average relative treatment effect: IPD-MA

We first synthesize the data using one-stage Bayesian hierarchical IPD-MA [35]. We will estimate the average relative treatment effect in terms of odds ratio (OR) for efficacy.

Let *y*_*ij*_ denote the dichotomous outcome of interest (*y*_*ij*_ = 1 for remission or low disease activity), for patient *i* where *i* = 1, 2, …, *n*_*j*_ in trial *j* out of *N* trials, *t*_*ij*_ be 0/1 for patient in control/intervention group, and *p*_*ij*_ is the probability of having the outcome.

$$ {y}_{ij}\sim \mathrm{Bernoulli}\left({p}_{ij}\right) $$

$$ \log \left(\frac{p_{ij}}{1-{p}_{ij}}\right)=\mathrm{logit}\left({p}_{ij}\right)={\alpha}_j+{\delta}_j{t}_{ij} $$

$$ {\delta}_j\sim N\left(\delta, {\tau}^2\right) $$

where *α*_*j*_ is the log odds of the outcome for the control group, in trial *j*, which is independent across trials; *δ*_*j*_ is the treatment effect (log OR), which we assume to be exchangeable across trials; *δ* is the summary estimate of the log-odds ratios for the intervention versus the control arm; and *τ*^2^ is the heterogeneity of *δ* across trials and normally distributed across trials.

#### Predicting treatment effect for patients with particular characteristics: a two-stage model

##### Data pre-processing

Within each study, the outcomes and the covariates will be evaluated for missing data, and we will further look at their distributional characteristics and correlations between the covariates (listed in the “At IPD level: for studies with IPD available” section). We will use multiple imputation methods for handling missing data [36]. We will consider data transformation for continuous variables to resolve skewness and re-categorization for categorical variables if necessary. If two or more variables are highly correlated, we will only retain the variable that is most commonly reported across studies and in the literature or the variable that has the least missing values.

##### Stage 1: Developing a baseline risk model

In this step, we will build a multivariable model to predict the probability that a patient, given her or his baseline characteristics, is likely to achieve remission or low disease activity irrespective of treatment; we will refer to this model as the *baseline risk model.* The risk model can be built using the patients from the control group only, or from both intervention and control group. The former is more intuitive; however, a simulation study indicated that models based on the whole participants produced estimates with narrower distribution of bias and were less prone to overfitting [37]. We will fit a multivariable logistic regression model:

$$ {y}_{ij}\sim \mathrm{Bernoulli}\left({r}_{ij}\right) $$

$$ \mathrm{logit}\left({r}_{ij}\right)={b}_{0j}+{\sum}_{k=1}^p{b}_{kj}\times {PF}_{ij k} $$

$$ {b}_{0j}\sim N\left({\beta}_0,{\sigma}_{\beta_0}^2\right) $$

$$ {b}_{kj}\sim N\left({\beta}_k,{\sigma}_{\beta_k}^2\right) $$

*r*_*ij*_ is the probability of the outcome for patient *i* from trial *j* at the baseline. *b*_0*j*_ is the intercept, which is exchangeable across studies. *PF*_*ijk*_ denotes the *k* prognostic factor (in total, there are *p* prognostic factors) in study *j* for patient *i*, and *b*_*kj*_ is the regression coefficient for *k* prognostic factor in study *j* and is exchangeable across studies.

In order to select the most appropriate model, we propose two approaches: (1) use previously identified prognostic factors and through discussions with rheumatologists to decide the subset of the most clinically relevant factors and estimate the coefficients using penalized maximum likelihood estimation shrinkage method and (2) use LASSO penalization methods for variable selection and coefficient shrinkage [38].

For each possible model, we will examine the sample size first, in order to assess the reliability of the model. We will calculate the events per variable (EPV) for our model, using all the categories of categorical variables and the degrees of freedom of continuous outcomes [39]. We will calculate efficient sample size for developing a logistic regression model [40]. Validation is essential in prediction model development. Since no external data is available, we can only use internal validation. Via resampling methods like bootstrap or cross-validation, we can estimate the calibration slope and *c*-statistic for each model, to indicate the ability of calibration and discrimination.

##### Stage 2: Developing a meta-regression model for treatment effects

We use the same notation system as that in the “Average relative treatment effect: IPD-MA” section. The logit(*r*_*ij*_) from stage 1 will be used as a covariate in the meta-regression model, both as a prognostic factor and as an effect modifier. Let ${\overline{\mathrm{logit}\left({r}_{ij}\right)}}^j$ denote the average of logit-risk for all the individuals in study *j*. The regression equation will be:

$$ {y}_{ij}\sim \mathrm{Bernoulli}\left({p}_{ij}\right) $$

$$ \mathrm{logit}\left({p}_{ij}\right)=\left\{\begin{array}{c}{a}_j+{g}_{0j}\times \left(\mathrm{logit}\left({r}_{ij}\right)-{\overline{\mathrm{logit}\left({r}_{ij}\right)}}^j\right), if\ {t}_{ij}=0\\ {}{a}_j+{\delta}_j+{g}_{0j}\times \left(\mathrm{logit}\left({r}_{ij}\right)-{\overline{\mathrm{logit}\left({r}_{ij}\right)}}^j\right)+{g}_j\times \left(\mathrm{logit}\left({r}_{ij}\right)-{\overline{\mathrm{logit}\left({r}_{ij}\right)}}^j\right), if\ {t}_{ij}=1\end{array}\right. $$

$$ {\delta}_j\sim N\left(\delta, {\tau}^2\right) $$

$$ {g}_{0j}\sim N\left({\gamma}_0,{\sigma}_{\gamma_0}^2\right) $$

$$ {g}_j\sim N\left(\gamma, {\sigma}_{\gamma}^2\right) $$

*a*_*j*_ is the log odds in the control group when a patient has a risk equal to the mean risk, which is assumed to be independent across trials. *g*_0*j*_ is the coefficient of the risk score, while *g*_*j*_ is the treatment effect modification of the risk score for the intervention group versus the control group; both are assumed to be exchangeable cross trials and normally distributed about a summary estimate *γ*_0_ and *γ* respectively.

##### Predicting the probability of having the outcome for a new patient

Assume a new patient *i* who is not from any trial *j* has a baseline risk score $\overset{\sim }{\mathrm{logit}\left({r}_i\right)}$ calculated from stage-one. In order to predict the absolute logit-probability to achieve the outcome, we use:

$$ \mathrm{logit}\left({p}_i\right)=a+{\gamma}_0\times \left(\overset{\sim }{\mathrm{logit}\left({r}_i\right)}-\overline{\mathrm{logit}(r)}\right), if{x}_i=0 $$

$$ \mathrm{logit}\left({p}_i\right)=a+\delta +{\gamma}_0\times \left(\overset{\sim }{\mathrm{logit}\left({r}_i\right)}-\overline{\mathrm{logit}(r)}\right)+\gamma \times \left(\overset{\sim }{\mathrm{logit}\left({r}_i\right)}-\overline{\mathrm{logit}(r)}\right), if{x}_i=1 $$

We would have estimated *δ*, *γ*_0_, and *γ* in the meta-regression stage. We will estimate $\overline{\mathrm{logit}(r)}$ as the mean of logit(*r*_*ij*_) across all the individuals and studies. For *a*, we will estimate it by synthesizing all the control arms. Then, we can calculate the individual probability of the outcome both for the control and the intervention and estimate the predicted absolute and relative treatment effect.

To evaluate the performance of the two-stage prediction model, we will use internally validation methods via both the traditional measures, like *c*-statistic, and measures relevant to clinical usefulness.

##### Publication bias

Considering that we will probably not be able to include all the relevant research works, as some studies or their results were likely not published owing to non-significant results (study publication bias and outcome reporting bias) [41, 42], we will evaluate this issue by comparing the search and screening results (as we will try to retrieve possibly unpublished reports) with the IPD we can get. If necessary, we will address it by adding the study’s variance as an extra covariate in the final IPD meta-regression model (see the section “Predicting treatment effect for patients with particular characteristics: a two-stage model”—“Stage 2: Developing a meta-regression model for treatment effects”).

##### Statistical software

We will use R for our analyses. Stage 2 will be performed in a Bayesian setting using R2Jags. For the development of the baseline risk model, we will use the pmsampsize command to estimate if the available sample size is enough. We will examine the linear relationship between each one of the prognostic factors and the outcome via rcs and anova commands. The LASSO model will be developed using the cv.glmnet command. We will use the lrm command for the predefined model based on prior knowledge, and then for the penalized maximum likelihood estimation, we will use the pentrace command. For the bootstrap internal validation (both for the baseline risk score and for the two-stage prediction model), we will use self-programmed R-routines.

## Discussion

We have presented the study protocol for a prediction model of treatment effects for RA patients receiving CTZ plus MTX, using a two-stage approach based on IPD-MA.

Though there are many optional drugs in treating RA, as treatment failure is relatively high, individualizing the treatment is imperative. Many prognostic models for RA have been proposed, but no one is sufficiently satisfactory [31]. We have discovered several problems.

Most previous models focused on long-term radiographic or functional prognosis. Although they are certainly the critical outcomes that both clinicians and patients care about, the complex therapeutic changes during the long treatment process are extremely difficult to handle in developing prediction models. Thus, it usually ends up with a simplified strategy, such as taking only the initial treatment into account, which compromises the clinical interpretation and relevancy of the model. On the other hand, a good short-term treatment response is always positively associated with good long-term prognosis [43, 44]. Predicting short-term treatment effect is instructive in clinical practice; however, research is lacking. A few established “short-term” disease-activity-oriented prediction models used an outcome measured at 6 months or 12 months. The problem is, unless in active-controlled studies, there would be considerable dropouts after 3–4 months; furthermore, due to ethnical issues, many trials would offer the non-responders other active treatments after 3–4 months. Under the ITT principle, patients were commonly analyzed as originally allocated; when dropouts were not negligible, imputation methods were usually used, but mostly single imputation such as non-responder imputation or last observation carried forward (LOCF) [45]. One may argue that these estimates were conservative to the intervention group though not precise. But in fact it is not always conservative for a *relative* effect estimate, while unbiased relative estimates are of critical interest in building personalized prediction models. As a result, in order to be methodologically rigorous, we choose the outcome measured at 3 months, when the randomization is likely kept, and which is consistent with the assessment time recommended by the guideline [1]. Additionally, thanks to the IPD, we will be able to use multiple imputation to handle missing data, rather than the single imputations used in primary RCTs.

We will use a two-stage approach to construct the prediction model using IPD-MA. Unlike the usual approach, which includes baseline features as prognostic factors and effect modifiers (through interaction terms) simultaneously, we first build a risk model for baseline factors, then treating the risk score as both a prognostic factor and an effect modifier. By doing so, overfitting problem caused by too many covariates and interaction terms can be alleviated. Moreover, since penalization will only be used in common regression during risk modeling stage but not in meta-regression, the compromised penalization in meta-regression can be avoided. For stage 1, generally there are two types of risk models. One is an *externally* developed model, which is derived based on data independent from the data used at stage 2, such as established models from previous studies, or using some other studies. The other is an *internally* developed risk model, for which the same data will be used to build both the risk model and the treatment effects model [37]. Because there is no well-established risk model to predict the short-term disease activity for RA patients and also because we will very probably not have sufficient sample size to divide the entire data into two parts, we will use the *internal* risk model for our study.

We acknowledge several limitations in our study. First, we handle effect modification at the level of risk scores, instead of particular covariates. That is, we will not try to identify specific effect modifiers. This may cause some problems in interpretation, as the concept of distinguishing prognostic factors and effect modifiers is well recognized. However, our approach assures the statistical power. Second, due to the restricted sample size, only internal validation is planned while external validation is lacking. It needs to be validated on an external dataset in the future. Third, we only focus on short-term treatment response for RA patients receiving two kinds of treatment, CTZ and MTX. Future studies may extend the scope to compare several kinds of therapies and treatment strategies and finally model for the long-term prognosis taking into consideration all the treatment processes.

## Supplementary information

**Additional file 1.** PRISMA-P checklist.

**Additional file 2.** Search Strategies.

## Data Availability

The data that support the findings of this study are available from http://vivli.org but restrictions apply to the availability of these data, which were used under license for the current study, and so are not publicly available. Data are however available from http://vivli.org upon reasonable request and application, after their permission.

## Abbreviations

ACR: American College of Rheumatology
AE: Adverse event
CDAI: Clinical Disease Activity Index
CTZ: Certolizumab
DAS28: Disease Activity Score based on the evaluation of 28 joints
DMARD: Disease-modifying anti-rheumatic drug (bDMARD, biologic DMARD; csDMARD, conventional synthetic DMARD; tsDMARDs, targeted synthetic DMARD)
EPV: Events per variable
EULAR: European League Against Rheumatism
FDA: Food and Drug Administration
IPD: Individual participant data
LOCF: Last observation carried forward
MA: Meta-analysis
MTX: Methotrexate
OR: Odds ratio
PF: Prognostic factor
RA: Rheumatoid arthritis
RCT: Randomized controlled trial
RoB: Risk of bias
SDAI: Simplified Disease Activity Index
TNF: Tumor necrosis factor
