# Supplementary material for: Predicting the treatment response of certolizumab for individual adult patients with rheumatoid arthritis: protocol for an individual participant data meta-analysis
Source: Syst Rev. 2020 Jun 12;9:140. doi: 10.1186/s13643-020-01401-x (PMC7477831; doi:10.1186/s13643-020-01401-x)
Supplement: Supplementary file 2 — Additional file 2. Search Strategies. [file 13643_2020_1401_MOESM2_ESM.doc]

**Appendix: Draft Search Strategies**

***A. Cochrane Library***

#1 MeSH descriptor: [Certolizumab Pegol] explode all trees

#2 Certolizumab or “Certolizumab Pegol” or Cimzia or CDP870 or CDP-870 or “CDP 870” or “CTZ”

#3 #1 or #2

#4 MeSH descriptor: [Arthritis, Rheumatoid] explode all trees

#5 Rheumatoid arthritis

#6 RA

#7 #4 or #5 or #6

#8 #3 and #7

***B. Scopus***

KEY((certolizumab OR "certolizumab pegol" OR cimzia OR cdp‐870 OR cdp870 OR "CDP 870" OR CTZ) AND ( "rheumatoid arthritis" OR RA )) AND NOT INDEX ( medline )

***C. Medline* via Ovid (Medline(R) and Medline(R) and Epub Ahead of Print, In-Process & Other Non-Indexed Citations, Daily and Versions(R))**

1 exp certolizumab/

2 (certolizumab or ‘certolizumab pegol’ or cimzia or CDP870 or CDP-870 or ‘CDP 870’ or CTZ).mp.

3 or/1-2

4 exp Arthritis, Rheumatoid/

5 ("Rheumatoid Arthritis" or RA or (Caplan$ and Syndrome?) or (Felty$ and S?ndrome) or (Rheumatoid and Nodule?) or (Sjogren$ and S?ndrome?) or (Sicca$ and S?ndrome?) or (Ankylos$ and Spondylit$) or (Spondylarthritis and Ankylopoietica) or (Rheumatoid$ and Spondylit$) or (Bechterew$ and Disease?) or (Mari-Struempell and Disease?) or (Adult and Onset and Still$ and Disease?)).mp.

6 or/4-5

7 3 and 6

8 exp randomized controlled trials/

9 exp double-blind method/

10 exp single-blind method/

11 exp cross-over studies/

12 randomized controlled trial.pt.

13 clinical trial.pt.

14 controlled clinical trial.pt.

15 (random$ adj2 control$ adj2 trial$).mp.

16 (clinic$ adj2 trial$).mp.

17 random$.mp.

18 placebo$.tw.

19 assign$.tw.

20 allocat$.tw.

21 (crossover$ or cross over$ or cross-over$).mp.

22 factorial$.tw.

23 ((singl$ or double$ or trebl$ or tripl$) adj (blind$ or mask$)).mp.

24 or/8-23

25 7 and 24
